# Hospital–Medicare Advantage vertical integration and medical loss ratios

**DOI:** 10.1093/haschl/qxag085

**Published:** 2026-04-06

**Authors:** Geronimo Bejarano, David J Meyers, Meredith B Rosenthal, Jeffrey Marr

**Affiliations:** Department of Health Services, Policy, and Practice, Brown University School of Public Health, Providence, RI 02903, United States; Department of Health Services, Policy, and Practice, Brown University School of Public Health, Providence, RI 02903, United States; Department of Health Policy and Management, Harvard School of Public Health, Boston, MA 02115, United States; Department of Health Services, Policy, and Practice, Brown University School of Public Health, Providence, RI 02903, United States

**Keywords:** Medicare Advantage, medical loss ratios, vertical integration

## Introduction

Health care in the United States continues to consolidate, including vertical integration (VI) between hospitals and insurers.^1^ Hospital–insurer VI may improve health outcomes through increased care coordination and reductions in utilization management from insurers for their vertically integrated providers, which increases access to care.^1^ However, VI also increases opportunities for strategic financial behaviors that find loopholes in existing regulations.^1-3^ For example, insurers are subject to minimum medical loss ratios (MLRs), with Medicare Advantage (MA) plans having to spend 85% of revenue on care.^4^ Through VI, insurers can artificially raise MLRs by internally transferring revenue through above-market rates to their provider subsidiaries, thereby inflating medical claims on patient care. This internal transfer would raise their MLR, even though the payments stay within the vertically integrated insurer-provider firm.^4^ In response, recent Department of Justice/Federal Trade Commission merger guidelines include recommendations on VI, where 2 firms on different levels of the supply chain merge.^5^ To better understand how VI may influence MLRs, we compared the MLRs between MA plans that are vertically integrated with hospitals and those that are nonintegrated.

## Methods

This cross-sectional study was exempt from informed consent due to use of deidentified data by the Brown University Institutional Review Board and was reported according to the STROBE (Strengthening the Reporting of Observational Studies in Epidemiology) guidelines. We used previously described data on hospital–MA plan VI that were identified using Agency for Healthcare Research and Quality (AHRQ) compendium files, public MA websites, and news reports.^1^ These data were merged with MLR data from the National Association of Insurance Commissioners compiled by Mark Farrah Associates at the parent organization level. The MLRs are calculated as the share of MA premiums that are used on claims. Parent organizations were considered nonintegrated if they are not vertically integrated with a hospital, hospital-owned if they are vertically integrated with a hospital and the parent organization is the hospital, or insurance partnership if they are vertically integrated with a hospital and the parent organization is the insurer. We described premiums, claims, and year of first VI by VI exposure. We analyzed MLRs from 2021 to 2024 by VI exposure (nonintegrated, hospital-owned, or insurance partnership) and year of first VI for hospital-owned parent organizations (nonintegrated, pre-2000, 2000–2015, or post-2015). All MLRs were weighted by the number of members. Data were analyzed from October to November 2025 using Stata, version 18.0.

## Results

Our sample included 173 MA plan parent organizations, where 113 are nonintegrated, 37 are hospital-owned, and 23 are insurance partnerships (Table 1). Hospital-owned organizations had, on average, the highest MLR (94.28%; SD: 6.68%) and lowest per-beneficiary premiums ($12 956.78; SD: $1808.72) and claims ($12 229.64; SD: $1984.52) compared with insurance partnership and nonintegrated parent organizations. Hospital-owned organizations were similarly represented in year of first VI, while insurance partnerships are growing (3 pre-2000, 9 in 2000–2025, and 11 post-2015).

**Table 1.** Characteristics of MA plan parent organizations (*n* = 173).

Variable	Hospital-owned (*n* = 37)	Insurance partnership (*n* = 23)	Nonintegrated (*n* = 113)
MLR, mean (SD)	94.28% (6.68%)	86.68% (2.90%)	89.74% (6.47%)
Premiums per beneficiary, average (SD)	$12 956.78 ($1808.72)	$14 982.11 ($1397.71)	$14 066.57 ($3962.95)
Claims per beneficiary, average (SD)	$12 229.64 ($1984.52)	$12 990.96 ($1327.24)	$12 536.94 ($3399.58)
Year of vertical integration			
Pre-2000	12	3	NA
2000–2015	12	9	NA
Post-2015	13	11	NA

Abbreviations: MA, Medicare Advantage; MLR, medical loss ratio; NA, not applicable.

Premiums, claims, and MLRs are weighted by member months. The MLR is calculated as the percentage of premium revenue that is used in claims, which is considered patient care. Insurers can also pay nonclaims payments (eg, bundled payments), which are not tied to any individual claim and therefore not included in these data. For vertically integrated MA parent organizations, they are considered hospital-owned if the parent organization is the hospital and they are considered insurance partnership if the parent organization is the insurer in public MA data.

**Figure 1.**
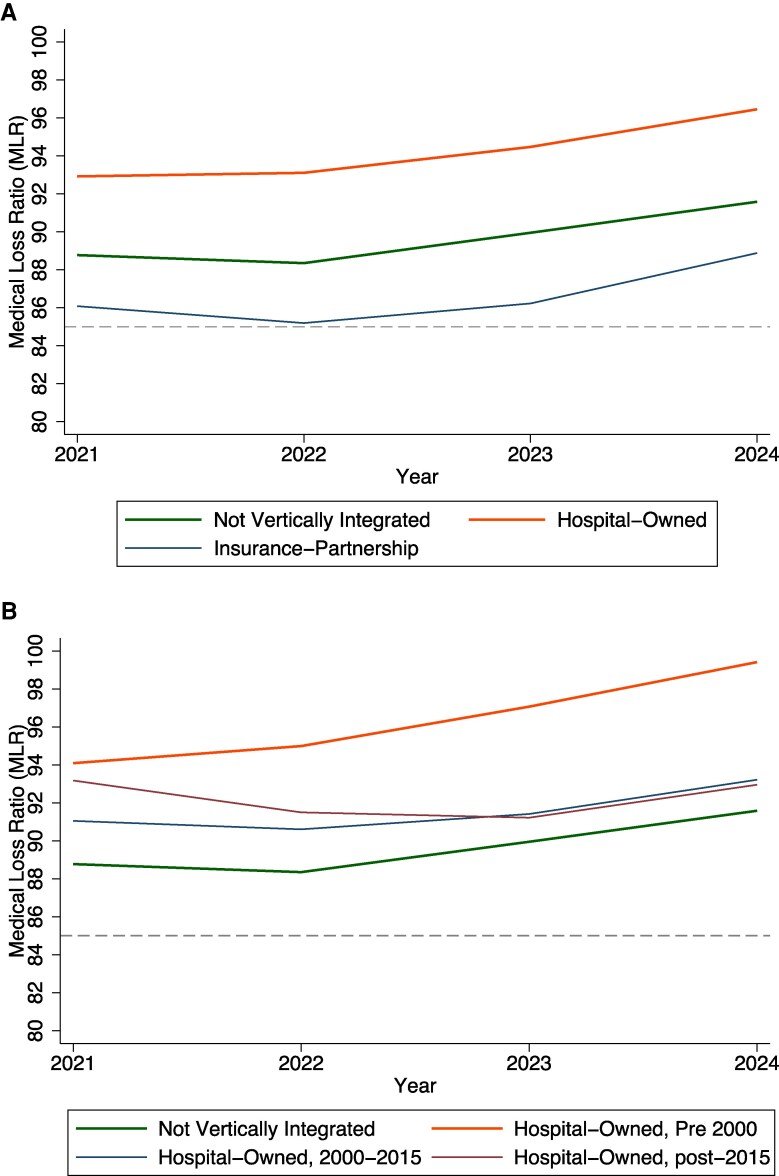
(A) Average medical loss ratios (MLRs) between 2021 and 2024 by type of parent organization vertical integration. (B) Average MLRs between 2021 and 2024 by year of first vertical integration for hospital-owned vertically integrated and nonintegrated parent organizations. The dashed line at 85% represents that MA plans have a requirement to have an MLR of at least 85% and are penalized if it is under that percentage. Hospital-owned and insurance partnerships were determined by the hospital–MA vertically integrated plan parent organization. In panel B, we did not include insurance partnerships as we cannot differentiate between their insurance partnership MA plans and the MA plans that do not have insurance partnerships within their parent organization. Medical loss ratios were weighted by member months. Abbreviation: MA, Medicare Advantage.

Medical loss ratios are increasing for each type of parent organization, with hospital-owned organizations consistently having the highest followed by nonintegrated then insurance partnerships (Figure 1). Among hospital-owned parent organizations, those first vertically integrated pre-2000 had the highest MLRs, while those vertically integrated post-2015 were higher in 2021 than those vertically integrated in 2000–2015, but the latter ranking was reversed by 2024.

## Discussion

Hospital-owned parent organizations have consistently higher MLRs than nonintegrated and insurance partnerships. A key concern with VI is that insurers may use payments to providers that they own to ensure that their MLRs are above the required threshold.^4^ The higher MLRs for hospital-owned parent organizations may suggest that they are transferring profits to their provider side. However, the lower claims and premiums by hospital-owned parent organizations suggest that differences may be also due to underlying markets, benefit design, or beneficiary mix rather than only internal transfers.

We found that parent organizations with the first VI pre-2000 have both the highest MLRs and the largest growth in MLR from 2021 to 2024 compared with insurance partnerships, non–vertically integrated, and hospital-owned parent organizations that vertically integrated later. Older VI parent organizations have also been found to have higher premiums and star ratings, suggesting being more established with higher resources compared with newer vertically integrated parent organizations, which may allow them the ability to fully develop internal pricing strategies with higher priced internal transfers leading to higher MLRs.^1^ This finding suggests that different types of vertically integrated firms (eg, hospital-owned or insurance partnership) may not pursue the same MLR strategies.

Our study is limited in that the MLRs are reported at the parent organization level while our measure of VI is at the contract level. In effect, we capture the impact of any VI among the contracts operated by a parent organization. Because only a subset of contracts in the parent organization may be vertically integrated, our study likely yields an underestimate of the association between VI and MLR for any given contract. Our data do not include nonclaims payments, which would increase the current MLRs. Additionally, the study data do not allow for controlling for other factors that may explain differences in MLRs, including beneficiary case mix, coding intensity, geographic market, benefit design, or care management strategies. However, prior work has found that VI MA contracts tend to have healthier patient populations who are less likely to be dual-eligible compared with those in nonintegrated MA plans.^1^ Antitrust agencies and policymakers are considering increasing transparency of ownership and lowering the merger price that triggers review. Our findings add to the literature that can inform current proposals by the Centers for Medicare and Medicaid Services and Federal Trade Commission in considering increased oversight of VI in health care through enhanced transparency requirements for payments to vertically integrated providers and adjusting MLRs to better reflect provider costs.^2,4,5^

## Supplementary Material

qxag085_Supplementary_Data

## Data Availability

The data used in this study is under a data use agreement and therefore cannot be made available. The code used in this study can be requested from the corresponding author.
